# Association of Maternal Mild Hypothyroidism With Offspring Neurodevelopment in TPOAb-Negative Women: A Prospective Cohort Study

**DOI:** 10.3389/fendo.2022.884851

**Published:** 2022-06-29

**Authors:** Qingru Wang, Yangqian Jiang, Hong Lv, Qun Lu, Shiyao Tao, Rui Qin, Lei Huang, Cong Liu, Xin Xu, Siyuan Lv, Mei Li, Zhi Li, Jiangbo Du, Yuan Lin, Hongxia Ma, Xia Chi, Zhibin Hu, Tao Jiang, Guoying Zhang

**Affiliations:** ^1^ State Key Laboratory of Reproductive Medicine, Nanjing Medical University, Nanjing, China; ^2^ Department of Epidemiology, Center for Global Health, School of Public Health, Nanjing Medical University, Nanjing, China; ^3^ State Key Laboratory of Reproductive Medicine (Suzhou Centre), The Affiliated Suzhou Hospital of Nanjing Medical University, Suzhou Municipal Hospital, Gusu School, Nanjing Medical University, Suzhou, China; ^4^ Department of Toxicology and Nutritional Science, School of Public Health, Nanjing Medical University, Nanjing, China; ^5^ Key Laboratory of Modern Toxicology of Ministry of Education, School of Public Health, Nanjing Medical University, Nanjing, China; ^6^ Department of Maternal, Child and Adolescent Health, School of Public Health, Nanjing Medical University, Nanjing, China; ^7^ Department of Child Health Care, Women’s Hospital of Nanjing Medical University, Nanjing Maternity and Child Health Care Hospital, Nanjing, China; ^8^ Department of Biostatistics, School of Public Health, Nanjing Medical University, Nanjing, China; ^9^ Department of Obstetrics, The First Affiliated Hospital of Nanjing Medical University, Nanjing Medical University, Nanjing, China

**Keywords:** thyroid hypofunction, pregnancy, neurodevelopment, infancy, cohort study

## Abstract

**Objectives:**

Adequate maternal thyroid hormone availability is crucial for fetal neurodevelopment, but the role of maternal mild hypothyroidism is not clear. We aim to investigate the association of maternal mild hypothyroidism with neurodevelopment in infants at 1 year of age among TPOAb-negative women.

**Methods:**

The present study was conducted within the Jiangsu Birth Cohort. A total of 793 mother–infant pairs were eligible for the present study. Maternal thyroid function was assessed by measuring serum thyroid-stimulating hormone, free thyroxine, and thyroid peroxidase antibodies. Neurodevelopment of infants was assessed by using the Bayley Scales of Infant and Toddler Development third edition screening test (Bayley-III screening test).

**Results:**

In the multivariate adjusted linear regression analyses, infants of women with subclinical hypothyroidism and isolated hypothyroxinemia were associated with decreased receptive communication scores (*β* = −0.68, *p* = 0.034) and decreased gross motor scores (*β* = −0.83, *p* = 0.008), respectively. Moreover, infants of women with high-normal TSH concentrations (3.0–4.0 mIU/L) and low FT4 concentrations were significantly associated with lower gross motor scores (*β* = −1.19, *p* = 0.032), while no differences were observed in infants when the mothers had a high-normal TSH concentration and normal FT4 levels.

**Conclusions:**

Maternal subclinical hypothyroidism is associated with decreased receptive communication scores in infants at 1 year of age. In addition, maternal TSH concentration greater than 4.0 mIU/L and maternal isolated hypothyroxinemia are associated with impaired gross motor ability of infants, especially in infants of women with high-normal TSH concentrations (3.0–4.0 mIU/L).

## Introduction

Maternal mild hypothyroidism is a common endocrine condition and occurs in 5%–18% of all pregnancies, depending on the definition used and population studied (1–3). Thyroid hormone is essential for optimum neurodevelopment of the fetus, acting on various stages of fetal neurological development, including neuronal migration, synaptogenesis, glial cell proliferation, and glial cell myelination (4, 5). Because the fetal thyroid gland is not functional until mid-gestation, the fetus predominantly depends on the supply of maternal thyroid hormone during that period (6). Therefore, maternal mild hypothyroidism may impair the neurodevelopment in offspring.

Results from animal studies have shown that shortage of thyroid hormone is associated with impaired brain development (7). Data from a large case–control study have demonstrated reduced scores on tests of intelligence, attention, and visual-motor performance at 8 years of age among children of mothers with untreated overt hypothyroidism (OH) during pregnancy compared to euthyroid controls (8, 9). Evidence from observational studies has demonstrated that maternal isolated hypothyroxinemia (IH) is predominantly associated with various types of neurodevelopmental disorders in children (10–12). However, the detrimental effects of maternal subclinical hypothyroidism (SCH) on fetal neurodevelopment remain less well established. Some studies have demonstrated that the offspring of women with SCH have decreased intelligence and motor ability or increased risk for delay in neurodevelopment (13–15), whereas others have shown conflicting results (10, 16, 17). This variation may be attributed to the different criteria for elevated thyroid-stimulating hormone (TSH) used in different studies (18).

Previous international guidelines have recommended using fixed upper limits of 2.5 mIU/L for the first trimester and 3.0 mIU/L for the second and third trimesters (1–3). Because studies have demonstrated that using these fixed upper limits in the 2011 guidelines results in overdiagnosis of SCH (19–22), the upper limit of TSH at 4.0 mIU/L for each trimester of pregnancy was advocated by the American Thyroid Association (ATA) 2017 guidelines (18). Moreover, only a few studies have investigated infant neurodevelopment among women with TSH concentrations between 3.0 and 4.0 mIU/L. Thus, the present study aimed to examine the association of maternal mild hypothyroidism with infant neurodevelopment, especially in women with high-normal TSH concentrations (3.0–4.0 mIU/L).

## Methods

### Study Design and Participants

This cohort study was embedded in the Jiangsu Birth Cohort (JBC), a population-based prospective and longitudinal study recruiting women who planned to receive assisted reproductive technology (ART) and who are at their first trimester of spontaneous pregnancy (SP) at clinics in Jiangsu, China (23). When infants reached 1 year of age, they were invited to hospitals for systematic medical examination. The present study included the mother–infant pairs if the maternal TSH, free thyroxine (FT4), and thyroid peroxidase antibody (TPOAb) had been measured during pregnancy. Only mother–infant pairs with infants who had neurodevelopment data at the age of 1 year were included. We excluded pairs if the mother had a pre-existing thyroid disorder, if the mother had treatment for a thyroid disorder or the maternal TPOAb was positive, and we further exclude pairs if the mother had elevated concentrations of FT4 or suppressed TSH. The detailed cohort design and data collection have been previously published (23). This study was approved by the institutional review board of Nanjing Medical University, China NJMUIRB (2017) 002. Written informed consent was obtained from all the participants or the infants’ parents or guardians.

The participants were divided into four groups according to maternal thyroid hormone levels as follows: euthyroidism (ET, normal concentration of TSH and FT4), overt hypothyroidism (OH, elevated concentration of TSH with low concentration of FT4), subclinical hypothyroidism (SCH, elevated concentration of TSH with normal concentration of FT4), and isolated hypothyroxinemia (IH, normal concentration of TSH and low concentration of FT4).

### Maternal Thyroid Parameters

Measures of thyroid functions including TSH, FT4, and TPOAb, were evaluated by electro-chemiluminescent microparticle immunoassays kits using the Architect system (Roche GmbH, Mannheim, Germany) in the Department of Clinical Laboratory in the Nanjing Maternity and Child Health Care Hospital. The intra-assay coefficients of variations were as follows: TSH < 5.3% and FT4 < 5.3%. The normal range for TSH was 0.2–4.0 mIU/L according to the ATA 2017 guidelines (18). The specific reference intervals (95% CI: 10.55–17.21 pmol/L) of FT4 for the study population were obtained according to the National Institute of Clinical Biochemistry (NACB) (24). TPOAb was considered positive at concentrations greater than 34 IU/ml.

### Assessment of Neurodevelopment

When infants reached approximately 1 year of age, their neurodevelopment was assessed with the Bayley Scales of Infant and Toddler Development third edition screening test (Bayley-III screening test) by standardized trained pediatricians or occupational therapists in the presence of a primary caregiver (25). This test uses a subset of items from the full-length Bayley-III Scales, which has been widely validated and extensively used to compare developmental outcomes across individuals worldwide (25). Following standard procedures, we administered the following five subscales of the Bayley-III screen test: cognition, receptive language, expressive language, fine motor, and gross motor. Each subscale consisted of a series of developmental play tasks, and the subscale-specific raw scores of completed items were then recorded.

In the present study, a series of approaches was adopted to ensure the validity and reliability of the infant neurodevelopmental evaluation. The evaluation environment was quiet and non-interfering. One developmental neuropsychologist was appointed to provide professional training of the standardized administration before the investigation. Additionally, with the informed consent of guardians, the entire assessments of all examiners were filmed, and some of the videos were randomly selected for secondary evaluation monthly. The pediatricians and occupational therapists who performed the tests were unaware of any other test outcomes, including maternal thyroid hormone levels during pregnancy.

### Covariates

Detailed information was obtained through questionnaires administered by trained interviews and comprised information on maternal and infant demographics, pregnancy-related information, and medical history. The covariates were chosen based on what was available within the data and following the literature review (18, 23). Potential confounding variables accounted for in the study were childbearing age (years) (26), maternal pre-pregnancy BMI (kg/m^2^) (27), parity (nulliparous/multiparous) (28), maternal education (<12/≥12 years) (29), mode of conception(spontaneous/ART) (30), diseases during pregnancy (diabetes/non-diabetes, and hypertension/non-hypertension) (31, 32), gestational age (33), and infant sex (male/female) (34).

### Statistical Analyses

Continuous variables with a normal distribution were expressed as mean (standard deviation, SD) and compared using Student´s *t*-test. Non-normally distributed variables were expressed as median (interquartile range, IQR) and compared using the Wilcoxon rank test. Categorical variables were expressed as percentage (%) and compared by the chi-squared test. Non-response analyses were performed to compare with the characteristics of the mother–infant pairs grouped by neurodevelopment assessment data availability according to different thyroid function groups.

Multivariate linear regression models were constructed to estimate the associations of maternal SCH, IH, and OH with offspring neurodevelopment. We developed a directed acyclic graph (DAG; **Figure S1**) to document our assumptions about the association between the covariates, exposure, and outcome, and specifically to hypothesize which variables were confounders (preceding both exposure and outcome). The casual directed acyclic graphs (DAGs) is a useful tool for researchers to understand the potential interplay among variables and to deduce which variables require control to minimize bias and which variables could introduce bias if controlled in the analysis (35). This DAG informed our staged modeling approach: model 1 was crude (unadjusted) and model 2 was adjusted for confounders (childbearing age, pre-pregnancy BMI, parity, maternal education level, mode of conception, and sex of infants). Gestational age at birth and diseases during pregnancy were considered potential intermediates and not included in the main model.

**Figure 1:**
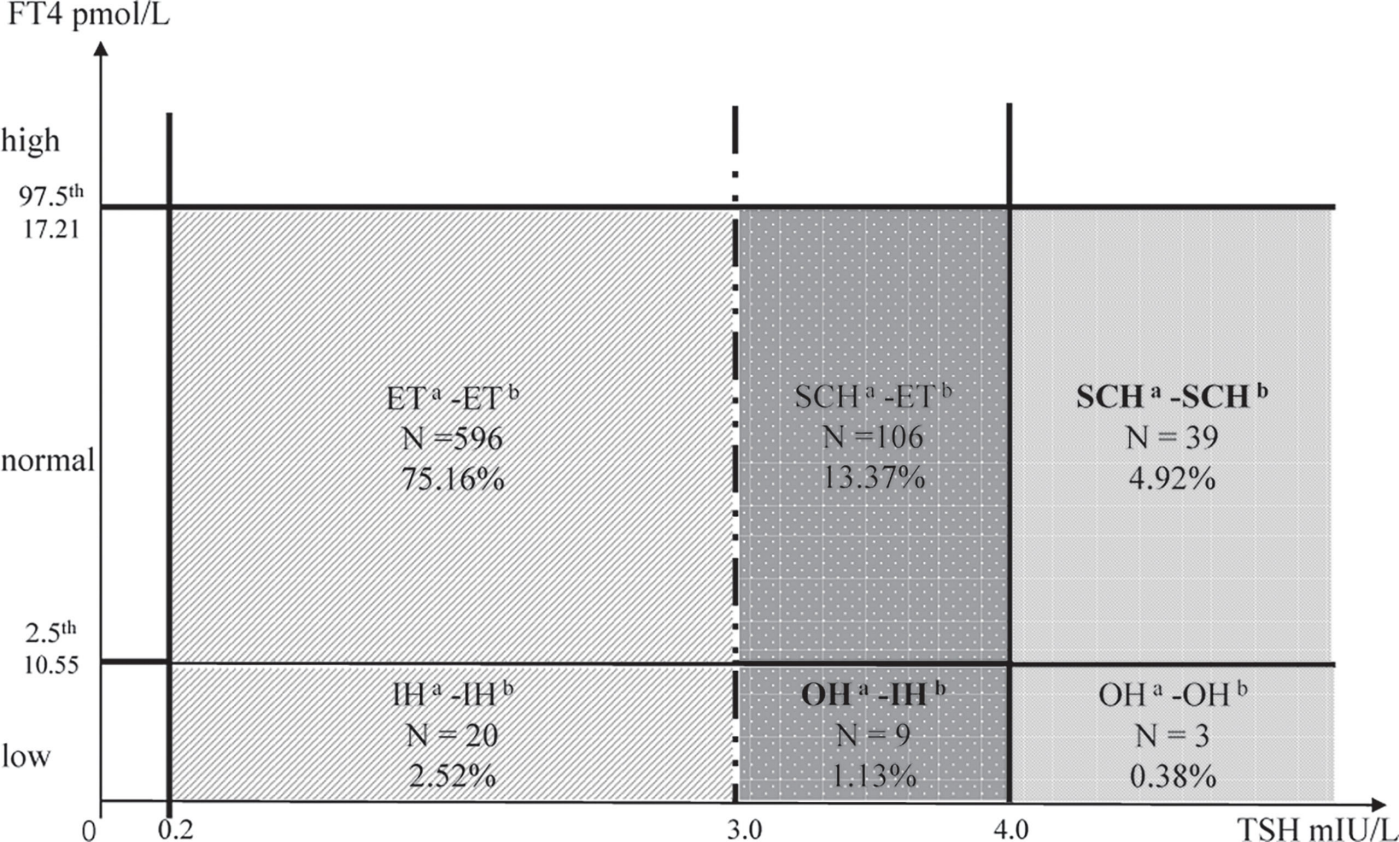
Thyroid function grouped on different TSH thresholds. ET, euthyroidism; SCH, subclinical hypothyroidism; OH, overt hypothyroidism; IH, isolated hypothyroxinemia. ^a^TSH thresholds at 3.0 mIU/L (2011 ATA guidelines). ^b^TSH thresholds at 4.0 mIU/L (2017 ATA guidelines).

Considering that the ATA 2011 guidelines define the upper limit for TSH at 3.0 mIU/L during mid-trimester (2), we further investigated the association of maternal mild hypothyroidism and infant neurodevelopment by different maternal TSH levels (0.2–3.0 vs. 3.0–4.0 mIU/L). The participants were divided into the following groups (**Figure 1**): Group ET^a^-ET^b^ (0.2–3.0 mIU/L TSH and normal FT4), Group SCH^a^-ET^b^ (3.0–4.0 mIU/L TSH and normal FT4), Group SCH^a^-SCH^b^ (TSH > 4.0 mIU/L and normal FT4), Group IH^a^-IH^b^ (0.2–3.0 mIU/L TSH and FT4 < 10.55 pmol/L), Group OH^a^-IH^b^ (TSH 3.0–4.0 mIU/L and FT4 < 10.55 pmol/L), and Group OH^a^-OH^b^ (TSH > 4.0 mIU/L and FT4 < 10.55 pmol/L)

We conducted two sensitivity analyses. First, we repeated our main analysis restricting to infants born at full term (37 weeks or greater), to examine whether adjusting for gestational age in our main models may have inadvertently introduced bias through unmeasured confounding between gestational age and infant neurodevelopment. Second, we repeated our main analysis in women without diseases during pregnancy, to examine whether associations differed.

Information on missing data is outlined in **Table 1**, and missing data on covariates were coded as a missing indicator for categorical variables and with median values for continuous variables in multivariable linear regression models. Two-sided *p* < 0.05 was considered statistically significant. All statistical analyses were conducted in R statistical software version 4.10.

**Table 1:** Characteristics of mother–infant pairs.

**Characteristics**	**ET (*n* = 702)**	**SCH (*n* = 39)**	**IH (*n* = 29)**	**OH (*n* = 3)**
TSH (mIU/L)	2.01 (1.43–2.60)	4.72 (4.26–5.45)**	2.07 (1.47–3.31)	5.48 (4.86–5.77)**
FT4 (pmol/L)	13.34 (12.30–14.40)	12.42 (11.81–13.94)*	10.15 (9.68–10.35)**	9.58 (9.54–10.01)**
Gestational age at blood sampling (weeks)	23.87 (0.67)	23.85 (0.50)	24.04 (0.56)	23.76 (0.59)
Childbearing age (years)	30.64 (3.89)	29.83 (4.49)	33.14 (4.00)**	31.40 (2.11)
Pre-pregnancy BMI (kg/m^2^), *n* (%)	21.63 (2.92)	20.79 (2.63)	22.94 (2.91)*	22.30 (3.55)
<18.5	76 (10.8)	7 (17.9)	1 (3.4)	0 (0.0)
18.5–23.9	487 (69.4)	24 (61.5)	17 (58.6)	2 (66.7)
24–27.9	110 (15.7)	6 (15.4)	10 (34.5)	1 (33.3)
≥28	23 (3.3)	0 (0.0)	1 (3.4)	0 (0.0)
Missing	6 (0.8)	2 (5.1)	0 (0.0)	0 (0.0)
Spontaneous conception, *n* (%)	559 (79.6)	30 (76.9)	17 (58.6)*	1 (33.3)
Primiparous, *n* (%)	546 (77.8)	30 (76.9)	21 (79.3)	3 (100.0)
Smoking during pregnancy, *n* (%)	0 (0.0)	0 (0.0)	1 (3.4)*	0 (0.0)
Drinking during pregnancy, *n* (%)				
Yes	4 (0.6)	2 (5.1)*	0 (0.0)	0 (0.0)
Missing	10 (1.4)	0 (0.0)	0 (0.0)	0 (0.0)
Maternal education (years), *n* (%)				
>12	614 (87.5)	30 (76.9)	20 (69.0)*	3 (100.0)
Missing	9 (1.3)	0 (0.0)	1 (3.4)	0 (0.0)
Diseases during pregnancy^a^				
Diabetes, *n* (%)	191 (27.2)	6 (15.4)	13 (44.8)	3 (100.0)*
Hypertension, *n* (%)	41 (5.8)	1 (2.6)	4 (13.8)	0 (0.0)
Vaginal delivery, *n* (%)	384 (54.7)	23 (59.0)	9 (31)*	1 (33.3)
Infant sex (female), *n* (%)	340 (48.4)	23 (59.0)	13 (44.8)	1 (33.3)
Birthweight (g)	3,411 (456)	3,407 (343)	3,365 (421)	4,107 (189)*
Gestational age (weeks)	39.46 (1.34)	39.72 (1.13)	39.26 (1.20)	39.48 (0.21)
Prematurity, *n* (%)	27 (3.8)	1 (2.6)	1 (3.4)	0 (0.0)
Duration of breastfeeding (months), *n* (%)				
>6	423 (60.3)	18 (46.2)	19 (65.5)	3 (100.0)
Missing	17 (2.4)	0 (0.0)	0 (0.0)	0 (0.0)
Age at Bayley-III screening test (days)	365.62 (6.56)	366.38 (6.67)	365.90 (6.99)	361.33 (0.58)

TSH, thyroid stimulation hormone; FT4, free thyroxine; ET, euthyroidism; SCH, subclinical hypothyroidism; OH, overt hypothyroidism; IH, isolated hypothyroxinemia; BMI, body mass index. Continuous variables are expressed as mean (SD) or median (IQR), whereas categorical variables are expressed as percentages. *p-value <0.05; **p-value <0.01. ^a^Diabetes includes chronic and gestational diabetes mellitus; hypertension includes chronic and pregnancy-induced hypertension.

## Results

**Figure 2:**
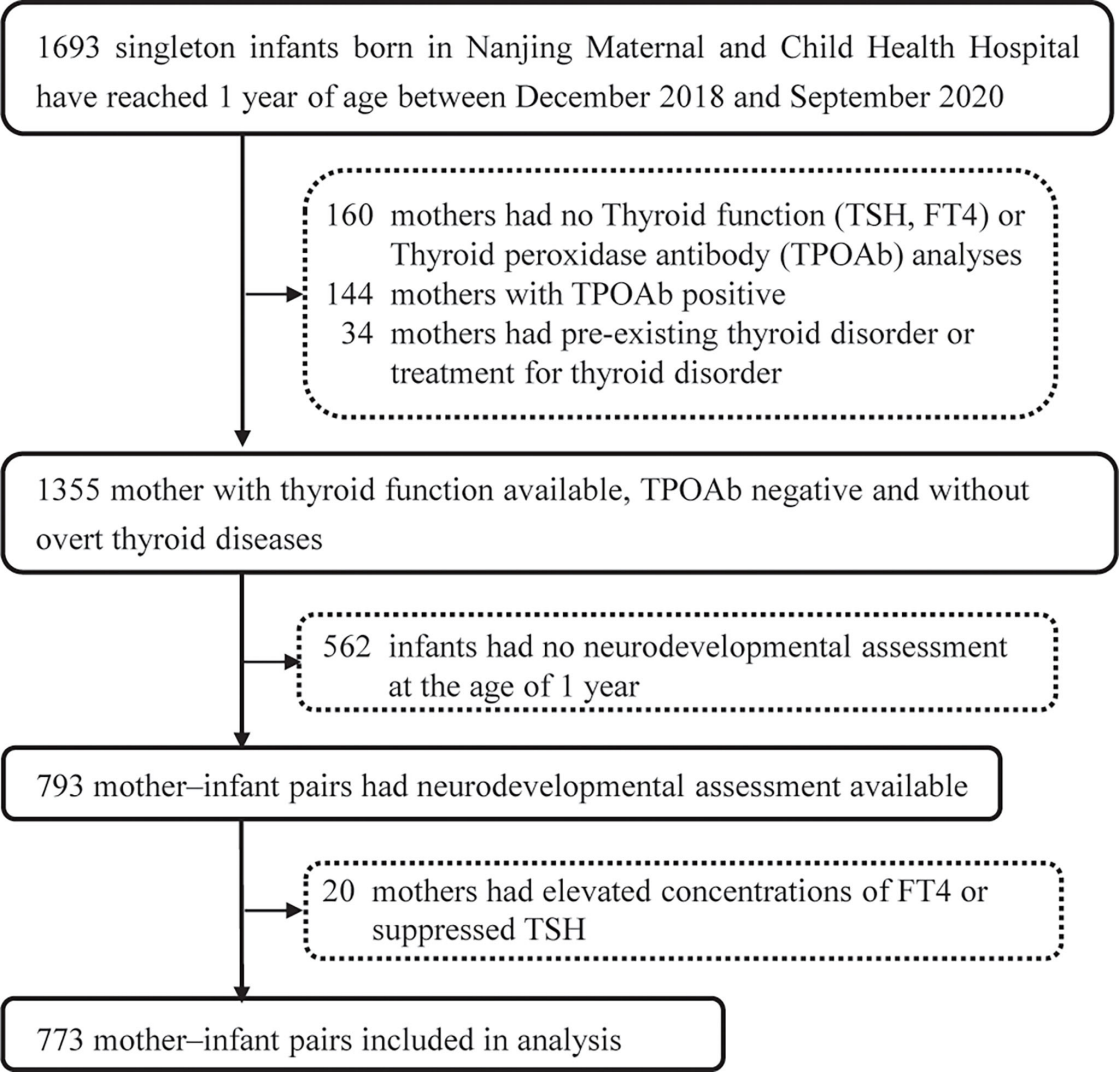
Flowchart of participants through the study. TSH, thyroid-stimulating hormone; FT4, free thyroxine.

From December 2018 to September 2020, a total of 1,693 singleton infants born in Nanjing Maternal and Child Health Hospital had reached 1 year old. Data on TSH, FT4 and TPOAb were obtained for 1,533 mothers. In total, 178 mother–infant pairs were excluded because they met exclusion criteria, and 562 mother–infant pairs were excluded because the infants did not have neurodevelopment assessment at the age of 1 year. Thus, the final study population was composed of 793 mother–infant pairs. Of these 793 mother–infant pairs, 4.92% of the women were identified as SCH, 3.65% of the women were identified as IH, and only 0.38% of the women were identified as OH according to the 2017 ATA guidelines; 2.52% (20/793) of the women who had elevated concentrations of FT4 or suppressed TSH were excluded (**Figure 2**). The remaining 773 pairs had 1.0%, 1.3%, 1.3%, and 2.2% missing values of pre-pregnancy BMI, drinking during pregnancy, maternal education, and duration of breastfeeding.

Compared to euthyroid women (ET group), the SCH group was more likely to have consumed alcohol during pregnancy, and the IH group was more likely to have higher childbearing age, higher pre-pregnancy BMI, and lower educational levels (**Table 1**). Comparisons of the characteristics of the mother–infant pairs grouped by neurodevelopment assessment data availability according to different groups showed no differences between participants and non-participants. However, the ET group of mother–infant pairs with available neurodevelopment data had higher mean pre-pregnancy BMI, and the SCH group of mother–infant pairs had lower FT4 concentration (**Table S1**)

Multivariate linear regression models showed that maternal SCH during pregnancy was associated with decreased receptive communication scores at 1 year of age (*β* = −0.68, *p* = 0.034). Infants exposed to maternal IH had decreased gross motor scores (*β* = −0.83, *p* = 0.008), while no significant difference was observed in infants of mothers with OH, which may be attributed to the small sample size (**Table 2**). When the 2011 ATA guidelines were used, similar results were obtained, and the infants exposed to maternal OH had lower gross motor scores (*β* = −1.11, *p* = 0.021) (**Table S2**). The stratified analysis results revealed that there was no statistically significant association of Group SCH^a^-ET^b^ (TSH 3.0–4.0 mIU/L and normal FT4) with infant neurodevelopment compared to Group ET^a^-ET^b^ (TSH 0.2–3.0 mIU/L and normal FT4), but Group SCH^a^-SCH^b^ (TSH >4.0 mIU/L and normal FT4) showed decreased receptive communication scores (*β* = −0.73, *p* = 0.025). Group IH^a^-IH^b^ (TSH 0.2–3.0 mIU/L and FT4 < 10.55 pmol/L) was associated with lower gross motor scores, but there was no significance after adjustment for potential confounders. However, Group OH^a^-IH^b^ (TSH 3.0–4.0 mIU/L and FT4 < 10.55 pmol/L) had lower gross motor scores (*β* = −1.19, *p* = 0.032) (**Table 3**). In the sensitivity analyses, we repeated our main analysis restricting to infants born at full term (37 weeks or greater) or in women without diseases during pregnancy, and the results were not significantly altered (**Tables S3, S4**).

**Table 2:** Association of maternal hypothyroidism with infant Bayley-III scores by the 2017 ATA guidelines.

Scores	*N*	Mean (SD)	Model 1			Model 2	
			β (95% CI)	*p*		β (95% CI)	*p*
**Cognition**							
ET	702	15.78 (2.09)	Ref			Ref	
SCH	39	15.74 (1.29)	−0.04 (−0.70, 0.62)	0.912		−0.09 (−0.75, 0.57)	0.788
IH	29	15.03 (1.97)	−0.75 (−1.51, 0.01)	0.055		−0.65 (−1.43, 0.12)	0.096
OH	3	16.33 (0.58)	0.55 (−1.77, 2.87)	0.641		0.51 (−1.82, 2.83)	0.670
**Receptive communication**							
ET	702	11.34 (2.01)	Ref			Ref	
SCH	39	10.74 (1.09)	−0.60 (−1.23, 0.03)	0.063		−0.68 (−1.31, −0.05)	0.034*
IH	29	10.83 (1.49)	−0.51 (−1.24, 0.21)	0.165		−0.35 (−1.09, 0.38)	0.347
OH	3	9.67 (1.53)	−1.68 (−3.89, 0.54)	0.139		−1.60 (−3.81, 0.62)	0.157
**Expressive communication**							
ET	702	12.09 (2.13)	Ref			Ref	
SCH	39	12.21 (1.98)	0.12 (−0.57, 0.80)	0.737		0.11 (−0.57, 0.80)	0.746
IH	29	11.97 (1.95)	−0.12 (−0.91, 0.66)	0.760		0.02 (−0.77, 0.82)	0.955
OH	3	12.33 (2.08)	0.25 (−2.16, 2.65)	0.842		0.41 (−1.99, 2.81)	0.739
**Fine motor**							
ET	702	13.11 (1.54)	Ref			Ref	
SCH	39	13.31 (1.56)	0.20 (−0.30, 0.69)	0.440		0.13 (−0.37, 0.62)	0.615
IH	29	12.86 (1.60)	−0.25 (−0.82, 0.33)	0.395		−0.09 (−0.67, 0.49)	0.760
OH	3	13.00 (1.00)	−0.11 (−1.86, 1.64)	0.901		0.01 (−1.74, 1.75)	0.993
**Gross motor**							
ET	702	14.56 (1.62)	Ref			Ref	
SCH	39	14.62 (1.68)	0.05 (−0.47, 0.58)	0.844		0.01 (−0.52, 0.53)	0.977
IH	29	13.25 (1.62)	−0.91 (−1.51, −0.30)	0.003**		−0.83 (−1.44, −0.22)	0.008**
OH	3	13.67 (2.89)	−0.90 (−2.74, 0.95)	0.341		−0.86 (−2.71, 0.98)	0.359

Model 1: crude. Model 2: adjusted for childbearing age, pre-pregnancy BMI, parity, mode of conception, maternal education, and sex of infants. CI, confidence interval; ET, euthyroidism; SCH, subclinical hypothyroidism; OH, overt hypothyroidism; IH, isolated hypothyroxinemia. *p-value <0.05; **p-value <0.01.

**Table 3:** Multivariable regression analysis to demonstrate the association of maternal TSH with infant Bayley-III scores.

Scores	*N*	Mean (SD)	Model 1			Model 2	
			β (95% CI)	*p*		β (95% CI)	*p*
**Cognition**							
ET^a^-ET^b^	596	15.77 (2.10)	Ref			Ref	
SCH^a^-ET^b^	106	15.82 (2.02)	0.05 (−0.38, 0.47)	0.827		0.06 (−0.36, 0.48)	0.786
SCH^a^-SCH^b^	39	15.74 (1.29)	−0.03 (−0.69, 0.63)	0.930		−0.08 (−0.75, 0.58)	0.810
IH^a^-IH^b^	20	15.30 (1.66)	−0.47 (−1.39, 0.44)	0.309		−0.35 (−1.27, 0.57)	0.456
OH^a^-IH^b^	9	14.44 (2.55)	−1.33 (−2.68, 0.02)	0.054		−1.32 (−2.69, 0.05)	0.060
OH^a^-OH^b^	3	16.33 (0.58)	0.56 (−1.76, 2.88)	0.637		0.51 (−1.82, 2.84)	0.668
**Receptive communication**							
ET^a^-ET^b^	596	11.39 (1.98)	Ref			Ref	
SCH^a^-ET^b^	106	11.09 (2.14)	−0.29 (−0.70, 0.11)	0.157		−0.29 (−0.70, 0.11)	0.154
SCH^a^-SCH^b^	39	10.74 (1.09)	−0.64 (−1.28, −0.01)	0.047*		−0.73 (−1.36, −0.09)	0.025*
IH^a^-IH^b^	20	10.85 (1.57)	−0.54 (−1.41, 0.33)	0.228		−0.43 (−1.31, 0.44)	0.335
OH^a^-IH^b^	9	10.78 (1.39)	−0.61 (−1.89, 0.68)	0.354		−0.32 (−1.62, 0.98)	0.631
OH^a^-OH^b^	3	9.67 (1.53)	−1.72 (−3.94, 0.50)	0.129		−1.64 (−3.86, 0.57)	0.147
**Expressive communication**							
ET^a^-ET^b^	596	12.09 (2.14)	Ref			Ref	
SCH^a^-ET^b^	106	12.07 (2.10)	−0.03 (−0.46, 0.41)	0.906		−0.03 (−0.47, 0.40)	0.883
SCH^a^-SCH^b^	39	12.21 (1.98)	0.11 (−0.57, 0.80)	0.747		0.11 (−0.58, 0.80)	0.757
IH^a^-IH^b^	20	12.30 (1.78)	0.21 (−0.74, 1.15)	0.666		0.35 (−0.60, 1.29)	0.476
OH^a^-IH^b^	9	11.22 (2.22)	−0.87 (−2.26, 0.52)	0.222		−0.73 (−2.14, 0.69)	0.314
OH^a^-OH^b^	3	12.33 (2.08)	0.24 (−2.16, 2.64)	0.844		0.4 (−2.01, 2.80)	0.746
**Fine motor**							
ET^a^-ET^b^	596	13.09 (1.53)	Ref			Ref	
SCH^a^-ET^b^	106	13.25 (1.62)	0.16 (−0.16, 0.48)	0.332		0.16 (−0.15, 0.48)	0.315
SCH^a^-SCH^b^	39	13.31 (1.56)	0.22 (−0.28, 0.72)	0.388		0.15 (−0.35, 0.65)	0.549
IH^a^-IH^b^	20	13.15 (1.79)	0.06 (−0.63, 0.75)	0.858		0.23 (−0.46, 0.92)	0.509
OH^a^-IH^b^	9	12.22 (0.83)	−0.87 (−1.88, 0.15)	0.096		−0.74 (−1.77, 0.28)	0.156
OH^a^-OH^b^	3	13.00 (1.00)	−0.09 (−1.84, 1.66)	0.922		0.03 (−1.72, 1.77)	0.977
**Gross motor**							
ET^a^-ET^b^	596	14.58 (1.58)	Ref			Ref	
SCH^a^-ET^b^	106	14.45 (1.85)	−0.13 (−0.47, 0.21)	0.450		−0.12 (−0.46, 0.21)	0.467
SCH^a^-SCH^b^	39	14.62 (1.68)	0.03 (−0.49, 0.56)	0.902		−0.01 (−0.54, 0.52)	0.969
IH^a^-IH^b^	20	13.80 (1.47)	−0.78 (−1.51, −0.06)	0.035*		−0.70 (−1.43, 0.03)	0.061
OH^a^-IH^b^	9	13.33 (1.32)	−1.25 (−2.32, −0.18)	0.022*		−1.19 (−2.28, −0.10)	0.032 *
OH^a^-OH^b^	3	13.67 (2.89)	−0.92 (−2.76, 0.93)	0.331		−0.89 (−2.73, 0.96)	0.348

Model 1: crude. Model 2: adjusted for childbearing age, pre-pregnancy BMI, parity, mode of conception, maternal education, and sex of infants. CI, confidence interval; ET, euthyroidism; SCH, subclinical hypothyroidism; OH, overt hypothyroidism; IH, isolated hypothyroxinemia. ^a^ TSH thresholds at 3.0 mIU/L (2011 ATA guidelines). ^b^ TSH thresholds at 4.0 mIU/L (2017 ATA guidelines). *p-value <0.05.

## Discussion

In this population-based prospective cohort study, we observed impaired neurodevelopment in infants prenatally exposed to maternal mild hypothyroidism. Interestingly, associations differed in magnitude by subtype of maternal mild hypothyroidism and domains of infant neurodevelopment. Infants of women with maternal SCH were associated with decreased receptive communication scores only with maternal TSH levels greater than 4.0 mIU/L. For gross motor ability, maternal IH was predominant, and the effect was mainly attributed to mothers with high-normal TSH levels (3.0–4.0 mIU/L). To the best of our knowledge, this is the first study to evaluate the association of maternal mild hypothyroidism with offspring neurodevelopment as stratified by maternal TSH concentration.

Our findings in this prospective cohort study agreed with previous results (13, 14). A retrospective study from China has demonstrated that the offspring of women with SCH (TSH > 4.21 mIU/L) tend to have lower mental development index (MDI) and psychomotor development index (PDI) scores (14). Similarly, a meta-analysis of 39 original articles, including 909,176 individuals, has shown that maternal SCH has distinctly higher risk of intellectual disability in offspring (36). In contrast, a retrospective study of the Danish National Birth Cohort has indicated no adverse association between SCH (TSH beyond 2.5 mIU/L), and offspring verbal IQ was found (10, 16). These findings indicate that the different TSH cutoff values may be important confounders in various studies, thereby underlining the importance of performing in-depth analyses of observed associations.

Our study identified a significant association between maternal SCH (TSH > 4.0 mIU/L) and decreased receptive communication score in infants, while no significance was observed when maternal TSH levels were greater than 3.0 mIU/L but within the normal range (TSH < 4.0 mIU/L). These findings confirmed that the use of 4.0 mIU/L as the cutoff for TSH avoids the potential risk of overdiagnosis in women with SCH, thereby strengthening the association between SCH during pregnancy and adverse neurodevelopment, mainly when the TSH level is greater than 4.0 mIU/L. In summary, our results further validated and enhanced the current body of evidence suggesting that SCH diagnosed by the ATA 2017 standards is appropriate for screening high-risk women.

We also observed that maternal IH was associated with lower gross motor score in infants. Because maternal OH increases the risk of motor neurodevelopmental delay (37), most studies have shown that IH during early pregnancy is associated with an increased risk of a delay in infant motor development (10, 38). Furthermore, findings from animal studies support the observed associations. Animal studies have demonstrated that the primary brain region affected by decreased availability of maternal FT4 includes the cerebellum, which plays a critical role in motor coordination and motor activity (39). However, the present study is the first to elucidate the association of high-normal TSH levels with offspring neurodevelopment. In the present study, maternal IH with TSH levels between 3.0 and 4.0 mIU/L resulted in significantly decreased gross motor scores in infants compared to infants of mothers with TSH levels lower than 3.0 mIU/L (1.19 vs. 0.70).

Clinical guidelines clearly indicate that OH in pregnant women should be treated (18). Although the above studies have reported adverse outcomes in children born to mothers with IH, no interventional data have yet been published demonstrating the beneficial effects of levothyroxine (LT4) therapy (17, 40). Additionally, a recent guideline from the American College of Obstetrics and Gynecology (ACOG) provides a more conservative approach, essentially advocating treatment only for OH (41). In the present study, among 29 women identified as IH, 9 of them were originally diagnosed as OH according to the ATA 2011 guidelines, indicating that they were previously advised to be treated but later were not. Therefore, these women deserve more attention considering the worse effects on neurodevelopment compared to those with TSH levels below 3.0 mIU/L. Additionally, the lack of treatment effects in large randomized clinical trials (RCTs) should be reviewed. Current RCTs lack stratification of TSH concentration, which may indicate that the treatment effect on the low-risk group (e.g., TSH < 2.5 or 3.0 mIU/L) is diluted, leading to the conclusion of no benefit of treatment. Therefore, further studies should be conducted to identify the optimal treatment threshold of TSH where the benefits of LT4 administration outweigh the risks.

The strengths of the present study included its population-based prospective design, the long follow-up period, and our findings were obtained from TPOAb-negative pregnant women. We observed that maternal SCH was associated with detrimental neurodevelopment of infants, even in women with negative tests for TPOAb, which provided robust evidence to support LT4 treatment of pregnant women with TSH levels ranging from 4.0 to 10.0 mIU/L independent of their thyroid autoantibody status (42). Although the Bayley-III screening test is a validated instrument, it is not a diagnostic tool, and relying on one informant for assessment of neurodevelopment is a major limitation. We did not collect data for thyroid hormone parameters of the offspring after birth, but studies have shown that maternal thyroid function with child neurodevelopment is not mediated or modified by differences in postnatal child thyroid function (43). In addition, the present study was conducted in a single center and had a small sample size. Therefore, our conclusion needs to be further confirmed by multicenter studies with large sample sizes.

## Conclusions

In summary, the present study demonstrated that maternal SCH is associated with decreased receptive communication scores in infants at 1 year of age. In addition, maternal TSH concentrations greater than 4.0 mIU/L and maternal IH are associated with impaired gross motor ability, especially in women with high-normal TSH concentrations (3.0–4.0 mIU/L). In addition, these findings suggest that clinicians should actively determine the primary cause of the decline in FT4 concentration for pregnant women in the higher end of the normal range (3.0–4.0 mIU/L) of TSH and low concentrations of FT4. Further studies are required to identify specific subgroups of women who may benefit from LT4 treatment.

## Data Availability Statement

The raw data supporting the conclusions of this article will be made available by the authors, without undue reservation.

## Ethics Statement

This study was approved by the institutional review board of Nanjing Medical University, China NJMUIRB (2017) 002. Written informed consent was obtained from all the participants or the infants' parents or guardians.

## Author Contributions

QW, YJ, and HL drafted the manuscript, analyzed the data, and interpreted the data. QW, TJ, and GZ designed the study, supervised the study, and critically revised the manuscript. All authors contributed to data collection, critically reviewed the article, and approved the final version to be published.

## Funding

This study was funded by the National Natural Science Foundation of China (81803305 and 82103854), the Natural Science Foundation of Jiangsu Province (BK20180683), and the Maternal and Child Health Association of Jiangsu Province (FYX202031).

## Conflict of Interest

The authors declare that the research was conducted in the absence of any commercial or financial relationships that could be construed as a potential conflict of interest.

## Publisher’s Note

All claims expressed in this article are solely those of the authors and do not necessarily represent those of their affiliated organizations, or those of the publisher, the editors and the reviewers. Any product that may be evaluated in this article, or claim that may be made by its manufacturer, is not guaranteed or endorsed by the publisher.
