# Supplementary material for: Association of Maternal Mild Hypothyroidism With Offspring Neurodevelopment in TPOAb-Negative Women: A Prospective Cohort Study
Source: Front Endocrinol (Lausanne). 2022 Jun 29;13:884851. doi: 10.3389/fendo.2022.884851 (PMC9278520; doi:10.3389/fendo.2022.884851)
Supplement: Supplementary file 1 [file DataSheet_1.docx]

Supplementary Material

# Supplementary Data

**Figure S1: Direct acyclic graph for offspring neurodevelopment.**


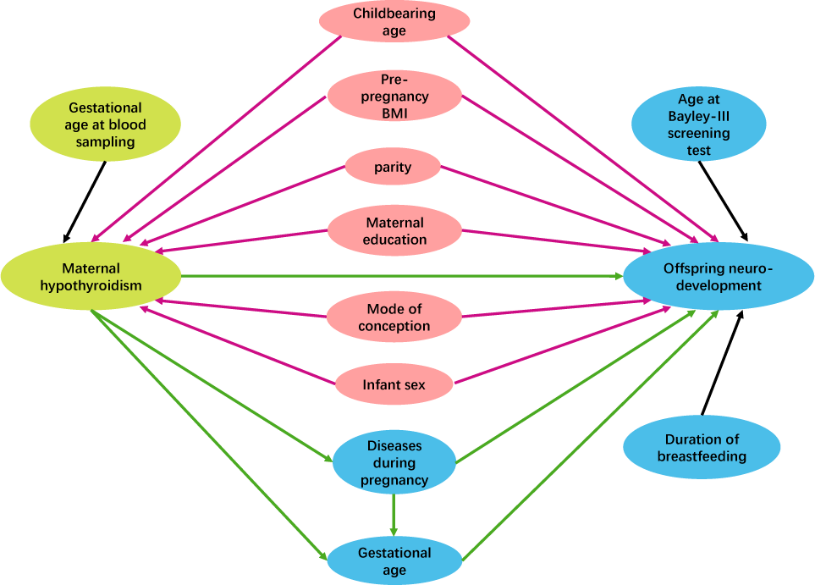


**Table S1: Characteristics of mother-infant pairs with and without Bayley-III screening test in different groups**

| **Characteristics** | **ET** | | |  | **SCH** | | |  | **IH+OH** | | |
| --- | --- | --- | --- | --- | --- | --- | --- | --- | --- | --- | --- |
|  | **with Bayley (n=702)** | **without Bayley (n=497)** | *P* |  | **with Bayley (n=39)** | **without Bayley (n=24)** | *P* |  | **with Bayley (n=32)** | **without Bayley (n=18)** | *P* |
| TSH (mIU/L) | 2.01 (1.43–2.60) | 2.01 (1.44–2.65) | 0.782 |  | 4.72 (4.26–5.45) | 4.43 (4.22–5.74) | 0.994 |  | 2.36 (1.51–3.41) | 2.47 (1.56–3.28) | 0.887 |
| FT4 (pmol/L) | 13.34 (12.30–14.40) | 13.41 (12.35–14.70) | 0.160 |  | 12.42 (11.81–13.94) | 13.68 (13.04–14.53) | 0.028* |  | 10.13 (9.62–10.35) | 9.96 (9.53–10.24) | 0.454 |
| Gestational age at blood sampling (weeks) | 23.87 (0.67) | 23.80 (0.66) | 0.076 |  | 23.85 (0.50) | 23.93 (0.68) | 0.619 |  | 24.01 (0.56) | 23.83 (0.62) | 0.301 |
| Childbearing age (years) | 30.64 (3.89) | 30.65 (3.79) | 0.937 |  | 29.83 (4.49) | 31.01 (3.00) | 0.262 |  | 32.98 (3.88) | 32.84 (5.16) | 0.919 |
| Pre-pregnancy BMI (kg/m^2^), n (%) | 21.63 (2.92) | 21.26 (2.82) | 0.026* |  | 20.79 (2.63) | 21.16 (3.78) | 0.648 |  | 22.88 (2.92) | 21.83 (1.69) | 0.170 |
| <18.5 | 76 (10.8) | 71 (14.3) | 0.202 |  | 7 (17.9) | 7 (29.2) | 0.196 |  | 1 (3.1) | 0 (0.0) | 0.336 |
| 18.5~23.9 | 487 (69.4) | 342 (68.8) |  |  | 24 (61.5) | 13 (54.2) |  |  | 19 (59.4) | 15 (83.3) |  |
| 24~27.9 | 110 (15.7) | 66 (13.3) |  |  | 6 (15.4) | 2 (8.3) |  |  | 11 (34.4) | 3 (16.7) |  |
| ≥28 | 23 (3.3) | 11 (2.2) |  |  | 0 (0.0) | 2 (8.3) |  |  | 1 (3.1) | 0 (0.0) |  |
| missing | 6 (0.8) | 7 (1.4) |  |  | 2 (5.1) | 0 (0.0) |  |  | 0 (0.0) | 0 (0.0) |  |
| Spontaneous conception, n (%) | 559 (79.6) | 392 (78.9) | 0.806 |  | 30 (76.9) | 20 (83.3) | 0.772 |  | 18 (56.2) | 10 (55.6) | 1.000 |
| Primiparous, n (%) | 546 (77.8) | 369 (74.2) | 0.178 |  | 30 (76.9) | 19 (79.2) | 1.000 |  | 26 (81.2) | 13 (72.2) | 0.701 |
| Smoking during pregnancy, n (%) |  |  | 0.243 |  |  |  | - |  |  |  | 1.000 |
| yes | 0 (0.0) | 1 (0.2) |  |  | 0 (0.0) | 0 (0.0) |  |  | 1 (3.1) | 0 (0.0) |  |
| missing | 0 (0.0) | 1 (0.2) |  |  | 0 (0.0) | 0 (0.0) |  |  | 0 (0.0) | 0 (0.0) |  |
| Drinking during pregnancy, n (%) |  |  | 0.886 |  |  |  | 0.698 |  |  |  | - |
| yes | 4 (0.6) | 4 (0.8) |  |  | 2 (5.1) | 0 (0.0) |  |  | 0 (0.0) | 0 (0.0) |  |
| missing | 10 (1.4) | 7 (1.4) |  |  | 0 (0.0) | 0 (0.0) |  |  | 0 (0.0) | 0 (0.0) |  |
| Maternal education (years), n (%) |  |  | 0.116 |  |  |  | 0.159 |  |  |  | 0.156 |
| >12 | 614 (87.5) | 421 (84.7) |  |  | 30 (76.9) | 21 (87.5) |  |  | 23 (71.9) | 17 (94.4) |  |
| missing | 9 (1.3) | 3 (0.6) |  |  | 0 (0.0) | 1 (4.2) |  |  | 1 (3.1) | 0 (0.0) |  |
| Diseases during pregnancy ^a^ |  |  |  |  |  |  |  |  |  |  |  |
| diabetes, n (%) | 191 (27.2) | 116 (23.3) | 0.149 |  | 6 (15.4) | 5 (20.8) | 0.832 |  | 16 (50.0) | 5 (27.8) | 0.219 |
| hypertension, n (%) | 41 (5.8) | 18 (3.6) | 0.106 |  | 1 (2.6) | 0 (0.0) | 1.000 |  | 4 (12.5) | 0 (0.0) | 0.307 |
| Vaginal delivery, n (%) | 384 (54.7) | 249 (50.1) | 0.130 |  | 23 (59.0) | 15 (62.5) | 0.990 |  | 10 (31.2) | 5 (27.8) | 1.000 |
| Fetal sex (female), n (%) | 340 (48.4) | 240 (48.3) | 1.000 |  | 23 (59.0) | 13 (54.2) | 0.911 |  | 14 (43.8) | 9 (50.0) | 0.897 |
| Birthweight (g) | 3,411 (456) | 3,365 (466) | 0.090 |  | 3,407 (343) | 3,442 (500) | 0.747 |  | 3,434 (459) | 3,528 (305) | 0.443 |
| Gestational age (weeks) | 39.46 (1.34) | 39.30 (1.68) | 0.060 |  | 39.72 (1.13) | 39.86 (1.50) | 0.682 |  | 39.28 (1.14) | 39.50 (0.85) | 0.486 |
| Prematurity, n (%) | 27 (3.8) | 28 (5.6) | 0.188 |  | 1 (2.6) | 1 (4.2) | 1.000 |  | 1 (3.1) | 0 (0.0) | 1.000 |

Abbreviations: TSH, thyroid stimulation hormone; FT4, free thyroxine; BMI, body mass index

Continuous variables are expressed as mean (SD) or median (IQR), whereas categorical variables are expressed as percentages

The P values demonstrated the differences between mother-infant pairs with and without Bayley-III screening test in different groups

**P* value <0.05

^a^ Diabetes includes chronic and gestational diabetes mellitus, hypertension includes chronic and pregnancy-induced hypertension

**Table S2: Association of maternal mild hypothyroidism with child Bayley-III scores by the 2011 ATA guidelines**

| **Scores** | **N** | **Mean (SD)** | **Model 1** | |  | **Model 2** | |
| --- | --- | --- | --- | --- | --- | --- | --- |
|  |  |  | **β (95% CI）** | ***P*** |  | **β (95% CI）** | ***P*** |
| **Cognition** | | | | | | | |
| ET | 596 | 15.77 (2.10) | Ref |  |  | Ref |  |
| SCH | 145 | 15.80 (1.85) | 0.03 (-0.35, 0.40) | 0.889 |  | 0.02 (-0.35, 0.39) | 0.913 |
| IH | 20 | 15.30 (1.66) | -0.47 (-1.39, 0.44) | 0.309 |  | -0.35 (-1.27, 0.57) | 0.454 |
| OH | 12 | 14.92 (2.35) | -0.86 (-2.03, 0.31) | 0.152 |  | -0.86 (-2.04, 0.33) | 0.159 |
| **Receptive communication** | | | | | | | |
| ET | 596 | 11.39 (1.98) | Ref |  |  | Ref |  |
| SCH | 145 | 11.00 (1.92) | -0.39 (-0.74, -0.03) | 0.033* |  | -0.41 (-0.76, -0.06) | 0.024* |
| IH | 20 | 10.85 (1.57) | -0.54 (-1.41, 0.33) | 0.228 |  | -0.43 (-1.31, 0.44) | 0.331 |
| OH | 12 | 10.50 (1.45) | -0.89 (-2.00, 0.23) | 0.120 |  | -0.66 (-1.79, 0.47) | 0.255 |
| **Expressive communication** | | | | | | | |
| ET | 596 | 12.09 (2.14) | Ref |  |  | Ref |  |
| SCH | 145 | 12.10 (2.06) | 0.01 (-0.37, 0.40) | 0.955 |  | 0.00 (-0.38, 0.39) | 0.981 |
| IH | 20 | 12.30 (1.78) | 0.21 (-0.74, 1.15) | 0.666 |  | 0.35 (-0.60, 1.30) | 0.474 |
| OH | 12 | 11.50 (2.15) | -0.59 (-1.8, 0.62) | 0.337 |  | -0.44 (-1.67, 0.78) | 0.481 |
| **Fine motor** | | | | | | | |
| ET | 596 | 13.09 (1.53) | Ref |  |  | Ref |  |
| SCH | 145 | 13.26 (1.60) | 0.17 (-0.11, 0.45) | 0.222 |  | 0.16 (-0.12, 0.44) | 0.261 |
| IH | 20 | 13.15 (1.79) | 0.06 (-0.62, 0.75) | 0.858 |  | 0.23 (-0.46, 0.92) | 0.508 |
| OH | 12 | 12.42 (0.90) | -0.67 (-1.55, 0.21) | 0.136 |  | -0.55 (-1.44, 0.34) | 0.228 |
| **Gross motor** | | | | | | | |
| ET | 596 | 14.58 (1.58) | Ref |  |  | Ref |  |
| SCH | 145 | 14.50 (1.80) | -0.09 (-0.38, 0.21) | 0.569 |  | -0.09 (-0.39, 0.20) | 0.531 |
| IH | 20 | 13.25 (1.62) | -0.78 (-1.51, -0.06) | 0.034* |  | -0.70 (-1.43, 0.03) | 0.061 |
| OH | 12 | 13.42 (1.68) | -1.17 (-2.09, -0.24) | 0.014* |  | -1.11 (-2.05, -0.17) | 0.021* |

Model 1: crude

Model 2: adjusted for childbearing age, pre-pregnancy BMI, parity, mode of conception, maternal education, sex of infants

Abbreviations: CI, confidence interval; ET, euthyroidism; SCH, subclinical hypothyroidism; OH, overt hypothyroidism; IH, isolated hypothyroxinemia

**P* value <0.05

**Table S3: Sensitivity analyses of maternal mild hypothyroidism with infant Bayley-III scores by the 2017 ATA guidelines**

| **Scores** | **N** | **Mean (SD)** | **Model 1** | |  | **Model 2** | |
| --- | --- | --- | --- | --- | --- | --- | --- |
|  |  |  | **β (95% CI）** | ***P*** |  | **β (95% CI）** | ***P*** |
| **Cognition** | | | | | | | |
| ET | 675 | 15.82 (2.08) |  |  |  |  |  |
| SCH | 38 | 15.79 (1.28) | -0.03 (-0.7, 0.64) | 0.927 |  | -0.07 (-0.74, 0.6) | 0.830 |
| IH | 28 | 15.00 (2.00) | -0.82 (-1.59, -0.05) | 0.038* |  | -0.73 (-1.52, 0.05) | 0.067 |
| OH | 3 | 16.33 (0.58) | 0.51 (-1.8, 2.83) | 0.665 |  | 0.46 (-1.86, 2.79) | 0.696 |
| **Receptive communication** | | | | | | | |
| ET | 675 | 11.37 (2.00) |  |  |  |  |  |
| SCH | 38 | 10.76 (1.10) | -0.61 (-1.25, 0.03) | 0.061 |  | -0.69 (-1.33, -0.05) | 0.034* |
| IH | 28 | 10.89 (1.47) | -0.48 (-1.22, 0.26) | 0.203 |  | -0.32 (-1.06, 0.43) | 0.402 |
| OH | 3 | 9.67 (1.53) | -1.71 (-3.91, 0.5) | 0.131 |  | -1.61 (-3.82, 0.6) | 0.155 |
| **Expressive communication** | | | | | | | |
| ET | 675 | 12.07 (2.10) |  |  |  |  |  |
| SCH | 38 | 12.24 (1.99) | 0.16 (-0.52, 0.85) | 0.640 |  | 0.15 (-0.53, 0.83) | 0.668 |
| IH | 28 | 12.07 (1.90) | 0 (-0.79, 0.79) | 0.995 |  | 0.14 (-0.66, 0.93) | 0.737 |
| OH | 3 | 12.33 (2.08) | 0.26 (-2.11, 2.63) | 0.830 |  | 0.42 (-1.95, 2.79) | 0.727 |
| **Fine motor** | | | | | | | |
| ET | 675 | 13.13 (1.56) |  |  |  |  |  |
| SCH | 38 | 13.34 (1.56) | 0.21 (-0.29, 0.72) | 0.409 |  | 0.16 (-0.35, 0.67) | 0.543 |
| IH | 28 | 12.89 (1.62) | -0.23 (-0.82, 0.35) | 0.435 |  | -0.09 (-0.68, 0.51) | 0.772 |
| OH | 3 | 13.00 (1.00) | -0.13 (-1.89, 1.64) | 0.888 |  | -0.01 (-1.78, 1.75) | 0.987 |
| **Gross motor** | | | | | | | |
| ET | 675 | 14.59 (1.63) |  |  |  |  |  |
| SCH | 38 | 14.71 (1.59) | 0.12 (-0.41, 0.66) | 0.648 |  | 0.08 (-0.45, 0.61) | 0.766 |
| IH | 28 | 13.25 (1.62) | -0.87 (-1.49, -0.26) | 0.006** |  | -0.78 (-1.4, -0.16) | 0.014* |
| OH | 3 | 13.67 (2.89) | -0.92 (-2.76, 0.92) | 0.328 |  | -0.88 (-2.73, 0.97) | 0.351 |

Model 1: crude

Model 2: adjusted for childbearing age, pre-pregnancy BMI, parity, mode of conception, maternal education, sex of infants

Abbreviations: CI, confidence interval; ET, euthyroidism; SCH, subclinical hypothyroidism; OH, overt hypothyroidism; IH, isolated hypothyroxinemia

**P* value <0.05; ***P* value <0.01

**Table S4: Sensitivity analyses of maternal mild hypothyroidism with infant Bayley-III scores by the 2017 ATA guidelines in women without diseases during pregnancy**

| **Scores** | **N** | **Mean (SD)** | **Model 1** | |  | **Model 2** | |
| --- | --- | --- | --- | --- | --- | --- | --- |
|  |  |  | **β (95% CI）** | ***P*** |  | **β (95% CI）** | ***P*** |
| **Cognition** | | | | | | | |
| ET | 483 | 15.82 (1.93) | Ref |  |  | Ref |  |
| SCH | 32 | 15.66 (1.29) | -0.17 (-0.85, 0.52) | 0.635 |  | -0.25 (-0.93, 0.44) | 0.481 |
| IH | 16 | 15.06 (2.46) | -0.76 (-1.71, 0.19) | 0.118 |  | -0.68 (-1.65, 0.28) | 0.167 |
| OH | 0 | - | - | - |  | - | - |
| **Receptive communication** | | | | | | | |
| ET | 483 | 11.44 (2.01) | Ref |  |  | Ref |  |
| SCH | 32 | 10.78 (1.10) | -0.66 (-1.36, 0.04) | 0.065 |  | -0.73 (-1.44, -0.02) | 0.044* |
| IH | 16 | 10.75 (1.53) | -0.69 (-1.66, 0.28) | 0.165 |  | -0.66 (-1.65, 0.34) | 0.197 |
| OH | 0 | - | - | - |  | - | - |
| **Expressive communication** | | | | | | | |
| ET | 483 | 12.12 (2.16) | Ref |  |  | Ref |  |
| SCH | 32 | 12.38 (2.01) | 0.25 (-0.51, 1.02) | 0.514 |  | 0.26 (-0.51, 1.03) | 0.507 |
| IH | 16 | 11.81 (1.72) | -0.31 (-1.37, 0.76) | 0.572 |  | -0.12 (-1.21, 0.97) | 0.828 |
| OH | 0 | - | - | - |  | - | - |
| **Fine motor** | | | | | | | |
| ET | 483 | 13.18 (1.52) | Ref |  |  | Ref |  |
| SCH | 32 | 13.16 (1.42) | -0.03 (-0.57, 0.51) | 0.919 |  | -0.12 (-0.66, 0.42) | 0.662 |
| IH | 16 | 12.88 (1.45) | -0.31 (-1.06, 0.44) | 0.421 |  | -0.17 (-0.93, 0.59) | 0.665 |
| OH | 0 | - | - | - |  | - | - |
| **Gross motor** | | | | | | | |
| ET | 483 | 14.66 (1.70) | Ref |  |  | Ref |  |
| SCH | 32 | 13.56 (1.41) | 0.08 (-0.5, 0.66) | 0.790 |  | 0.02 (-0.57, 0.6) | 0.959 |
| IH | 16 | 13.25 (1.62) | -1.02 (-1.82, -0.21) | 0.014* |  | -0.95 (-1.77, -0.13) | 0.023* |
| OH | 0 | - | - | - |  | - | - |

Model 1: crude

Model 2: adjusted for childbearing age, pre-pregnancy BMI, parity, mode of conception, maternal education, sex of infants

Abbreviations: CI, confidence interval; ET, euthyroidism; SCH, subclinical hypothyroidism; OH, overt hypothyroidism; IH, isolated hypothyroxinemia

**P* value <0.05
